# Comparison of Japanese Centenarians’ and Noncentenarians’ Medical Expenditures in the Last Year of Life

**DOI:** 10.1001/jamanetworkopen.2021.31884

**Published:** 2021-11-05

**Authors:** Yasuhiro Nakanishi, Yukio Tsugihashi, Manabu Akahane, Tatsuya Noda, Yuichi Nishioka, Tomoya Myojin, Shinichiro Kubo, Tsuneyuki Higashino, Naoko Okuda, Jean-Marie Robine, Tomoaki Imamura

**Affiliations:** 1 Department of Health and Welfare Services, National Institute of Public Health, Wako, Saitama, Japan; 2 Department of Public Health, Health Management and Policy, Nara Medical University, Kashihara, Nara, Japan; 3 Healthcare and Wellness Division, Mitsubishi Research Institute Inc, Chiyoda, Tokyo, Japan; 4 Japan Medical Association Research Institute, Tokyo, Japan; 5 Mécanismes Moléculaires Dans les Démences, École Pratique des Hautes Études, Institut National de la Santé et de la Recherche Médicale, University of Montpellier, Montpellier, France, and Paris Sciences & Lettres Research University, Montpellier, France; 6 Centre de Recherche Médecine, Sciences, Santé, Santé Mentale, Société, Centre National de la Recherche Scientifique, Institut National de la Santé et de la Recherche Médicale, Ecole des Hautes Études en Sciences Sociales, University of Paris, Paris, France

## Abstract

**Importance:**

Although research has shown that centenarians tend to experience shorter periods of serious illness compared with other age groups, few studies have focused on the medical expenditures of centenarians as a potential indicator of the scale of medical resources used in their last year of life.

**Objective:**

To compare Japanese centenarians’ and noncentenarians’ monthly medical expenditures during the year before death according to age and sex.

**Design, Setting, and Participants:**

This retrospective cohort study used linked national health and long-term care insurance data collected from April 2013 to March 2018 in Nara Prefecture, Japan, for residents aged 75 years or older who were insured under the Medical Care System for older adults and died between April 2014 and March 2018. Data were analyzed from April 2013 to March 2018.

**Exposures:**

Age of 100 years or older (centenarians) vs 75 to 99 years (noncentenarians).

**Main Outcomes and Measures:**

The numbers of unique inpatients and outpatients and medical expenditures related to decedents’ hospitalization and outpatient care were extracted and analyzed based on sex and age group. The Jonckheere-Terpstra test was used to identify trends in unadjusted medical expenditures by age group, and generalized estimating equations were used to estimate monthly median expenditures by age group with adjustment for comorbidity burden and functional status.

**Results:**

Of 34 317 patients aged 75 to 109 years (16 202 men [47.2%] and 18 115 women [52.8%]) who died between April 2014 and March 2018, 872 (2.5%) were aged 100 to 104 years (131 men [15.0%] and 741 women [85.0%]) and 78 (0.2%) were aged 105 to 109 years (fewer than 10 were men). The analysis of unadjusted medical expenditures in the last year of life showed a significant trend of lower expenditures for the older age groups; the median adjusted total expenditures during the 30 days before death by age group were $6784 (IQR, $4884-$9703) for ages 75 to 79 years, $5894 (IQR, $4292-$8536) for 80 to 84 years, $5069 (IQR, $3676-$7150) for 85 to 89 years, $4205 (IQR, $3085-$5914) for 90 to 94 years, $3522 (IQR, $2626-$4861) for 95 to 99 years, $2898 (IQR, $2241-$3835) for 100 to 104 years, and $2626 (IQR, $1938-$3527) for 105 to 109 years. The proportion of inpatients among all patients in the year before death also decreased with increasing age: 4311 of all 4551 patients aged 75 to 79 years (94.7%); 43 of all 78 patients aged 105 to 109 years (55.1%); 2831 of 2956 men aged 75 to 79 years (95.8%); 50.0% of men aged 105 to 109 years (the number is not reported owing to the small sample size); 1480 of 1595 women aged 75 to 79 years (92.8%); and 55.7% of women aged 105 to 109 years (the number of women is not reported to prevent back-calculation of the number of men). Specifically, 274 of 872 patients aged 100 to 104 years (31.4%) and 35 of 78 patients aged 105 to 109 years (44.9%) had not been admitted to a hospital in the year before death.

**Conclusions and Relevance:**

This cohort study found that medical expenditures in the last year of life tended to be lower for centenarians than for noncentenarians aged 75 years or older in Japan. The proportion of inpatients also decreased with increasing age. These findings may inform future health care services coverage and policies for centenarians.

## Key Points

**Question:**

Are there differences in Japanese centenarians’ and noncentenarians’ medical expenditures in the year before death according to age and sex?

**Findings:**

In this cohort study of 34 317 Japanese patients aged 75 years or older, in the last year of life, medical expenditures and the proportion of inpatients were significantly lower in older age groups, with the lowest values among patients aged 105 to 109 years.

**Meaning:**

The findings suggest that health care expenses in the last year of life may be lower for centenarians than for noncentenarians.

## Teaser

This cohort study examines differences in medical expenditures in the year before death among Japanese centenarians and noncentenarians aged 75 years or older by age and sex.

## Introduction

In recent years, the numbers of centenarians (persons aged 100 years or older), semi-supercentenarians (persons aged 105 to 109 years), and supercentenarians (persons aged 110 years or older) have been increasing, mainly in advanced industrialized nations including Japan.^1,2,3^ According to data published by the United Nations,^4^ as of 2018, the estimated number of centenarians worldwide was approximately 500 000, with the largest number (83 000) in the US, followed by Japan (69 000), China (61 000), and India (42 000). Japan has the highest proportion of centenarians among the general population, with 53.9 centenarians per 100 000 people, followed by France (28.9 per 100 000 people), Italy (26.7 per 100 000 people), and the US (25.3 per 100 000 people). As of August 2021, the oldest known living person in the world was a Japanese woman, Kane Tanaka.^5^ She was aged 117 years and 41 days when her age was verified by Guinness World Records on February 12, 2020,^6^ and she turned 118 years of age on January 2, 2021.^7^ Jeanne Louise Calment (1875-1997) from France holds the current record for the longest female life span (122 years and 164 days).^8^ The current record for the longest male life span is 116 years and 54 days, held by Jiroemon Kimura (1897-2013) from Japan.^9^

Although centenarians generally have higher rates of disability compared with noncentenarians,^10,11^ previous studies have indicated that compared with other age groups, centenarians tend to experience shorter periods of serious illness^12,13,14,15,16,17^; this trend has been especially pronounced among supercentenarians.^18^ These findings are consistent with the compression-of-morbidity hypothesis by Fries^19^ and suggest that an individual’s ability to remain healthy is associated with shorter duration of morbidity and disability toward the end of life and decreased overall lifetime disability.^14^ Studies have also indicated that a shorter duration of disability (although not necessarily morbidity) is an essential requirement for survival to exceptionally old age.^17,20^ The finding that supercentenarians’ disability and sometimes morbidity are shorter suggests that supercentenarians may be approaching the limit of the human life span,^15^ an idea that has influenced demographic studies examining centenarians and the human life span.^21,22,23,24,25,26,27^ The biological mechanisms that might limit the human life span remain unclear,^28^ and there are contrasting viewpoints on the subject. For example, a 2016 study by Dong et al^27^ claimed that the human life span is limited, whereas in 2018, Barbi et al^24^ denied the existence of such a limit. Both studies have attracted considerable attention.^29,30^

It is expected that centenarians would have shorter periods of critical health conditions and lower premortem medical expenditures than would noncentenarians.^13,31^ Previous studies on health economics have revealed that as older adult patients’ age increases, their medical expenditures decrease during the 1 to 3 years before death.^32,33,34,35,36,37,38,39,40,41,42,43,44,45,46^ This trend has been consistent across countries with differing health care systems, and the same trend has been reported in Japan.^40,41,42^ However, to our knowledge, only a few studies^43,44,45,46^ have reported on the premortem medical expenditures of centenarians. A study^43^ analyzing UK electronic health records between 2010 and 2014 reported the mean annual predicted health care costs in the last year of life by age for patients up to age 105 years but not by sex. Another study^44^ analyzed US data from 2000 to 2011 from a 5% sample of claims for Medicare-covered services under Parts A, B, and D from the Chronic Conditions Data Warehouse of the Centers for Medicare & Medicaid Services; the study showed the Medicare spending per capita among people aged 100 years or older who died in 2011 but not by sex, and a specific figure of this spending was reported only for people aged 100 years. Other previous studies regarding premortem medical expenditures^32,33,34,35,36,37,38,39,40,41,42^ grouped participants who were older than 84, 89, or 94 years. Thus, they did not specifically consider the premortem medical expenditures of centenarians. The typical monthly medical expenditures incurred by centenarians during the 1 year before death by age and sex are still unknown. Therefore, this study aimed to examine Japanese centenarians’ medical expenditures in the year before death, assess the scale of medical resources used, and compare these individuals’ expenditures with those of noncentenarians by age and sex.

## Methods

### Design

This population-based retrospective cohort study was conducted using linked national health and long-term care (LTC) insurance claims data in Nara Prefecture, located in the south-central region of Japan’s main island. Ethics approval was obtained from Nara Medical University. The requirement for informed consent was waived because of the anonymous nature of the claims data sets; the data sets generated during the current study complied with the publication policy of Nara Prefecture. Data were analyzed from April 2013 to March 2018. This study followed the Strengthening the Reporting of Observational Studies in Epidemiology (STROBE) reporting guideline.

### Public Medical System for Patients Aged 75 Years or Older in Japan

In Japan, universal health insurance coverage was established in 1961 and reformed in 2008 to address the increasing costs associated with the growing older adult population.^47^ The system is designed for patients to switch to the Medical Care System for older adults (aged 75 years or older) once they reach this age. The insurance system is managed at the prefectural level, with 50% of medical costs paid by public funds, 40% by the working population (through health insurance premiums), and 10% by the older population aged 75 years or older.^48^ In 2013, the national insurance covered approximately 99% of the 170 000 people aged 75 years or older in Nara Prefecture.

### Participants

This study enrolled patients insured under the Medical Care System for adults aged 75 years or older between April 2013 and March 2018. Health and LTC insurance claims data were obtained for the residents of Nara Prefecture who were aged 75 years or older^49^ and linked using a unique identifier of each insured patient as the linkage key. Data on residents who died between April 2014 and March 2018 were extracted and targeted for analysis. However, for residents who had died at the age of 75 years, it was not possible to trace the insurance claims data for a full year; thus, these residents were excluded from the sample. Decedents whose data were extracted were classified as those whose insurance expiration date and date of last recorded medical procedures and costs were the same and those for whom these dates were separated by 1 day. This classification allowed us to identify patients who received health care services coverage just before death.

According to the Japanese national census conducted every 5 years, the prefectural population was 1.36 million in 2015, with a population density of 370 persons per square kilometer. The population of adults aged 65 years or older in the prefecture was 388 614 (28.7% of the prefectural population), and its population of adults aged 75 years or older was 180 549 (13.3%). The adults aged 75 years or older in the prefecture included 667 younger centenarians (aged 100 to 104 years), 47 semi-supercentenarians (aged 105 to 109 years), and 1 supercentenarian (110 years or older). For reference, the 2015 population in Japan was 127 million, and the population density in Japan was 341 persons per square kilometer. Overall, 26.6% of the total population in Japan consisted of adults aged 65 years or older and 12.8% consisted of adults aged 75 years or older; specifically, there were 57 874 younger centenarians, 3770 semi-supercentenarians, and 146 supercentenarians.

### Outcomes

This study investigated the numbers of unique inpatients and outpatients and the decedents’ relevant medical expenditures for hospitalization and outpatient care. We calculated the hospitalization expenditures using hospital and combined diagnostic procedures claims and the outpatient expenditures using the outpatient treatment and dispensed prescription medication claims. Food and living care costs incurred during hospitalization were included as hospitalization expenditures. Home medical care costs were included as outpatient expenditures, but dental care costs were excluded.^50,51^

Decedents’ medical expenditures for hospitalization and outpatient care were calculated by sex and age group (5-year categories). The proportion of inpatients among all patients by sex and age group in the 1 year before death was calculated by dividing the number of patients with hospitalization expenditures by the number of unique patients with hospitalization and/or outpatient expenditures.

We also validated the number of deaths by referring to the official statistics from the Ministry of Health, Labour and Welfare.^52^ In addition, we calculated the median hospitalization- and non–hospitalization-related medical expenditures incurred and the proportion of inpatients among all patients every 30 days during the last year before each target patient’s date of death, and we compared these in each period according to the 5-year age groups. All medical expenditures were converted at an exchange rate of ¥120 to $1.00, the approximate average exchange rate in 2015.^53^

### Statistical Analysis

Descriptive analyses were conducted to obtain the number of patients and medical expenditures. The Jonckheere-Terpstra test was used to assess the trends in medical expenditures by age group. This test is a nonparametric, rank-based trend test for continuous variables, and it generates a standardized statistic that shows the strength of trends for parameters to decrease or increase across groups.^54,55,56,57^

To estimate median monthly medical expenditures in the year before death by controlling for confounders, such as comorbidity burden and functional status, generalized estimating equations with a log-link function, a gamma distribution, an unstructured correlation, and a model-based estimate were used.^40,58,59,60,61^ The dependent variables were the amount of total medical expenditures, hospitalization expenditures, and outpatient expenditures during each 30-day period in the 1 year before death, and the independent variables were sex, age group, diagnosis-related codes based on the *International Statistical Classification of Diseases and Related Health Problems, Tenth Revision* 3-character code block categories^62^ (the top 200 most frequent codes without a suspected disease flag among targeted decedents were applied as independent variables and are shown in eTable 1 in the Supplement), and care-needs levels (categorized into 2 levels of support needs or 5 levels of care needs). In the Japanese public LTC insurance system, all residents aged 40 years or older pay insurance premiums, and those aged 65 years or older (and those aged 40 to 64 years who have 1 of 16 specified diseases such as cancer or rheumatoid arthritis) are eligible for benefits. Municipalities are the insurers, and an applicant’s eligibility for LTC insurance benefits is assessed by a questionnaire regarding current physical and mental status (74 items), with a preliminary categorization by a computer algorithm into 1 of 7 levels of support and care needs. This assessment is then reviewed and finalized by an expert committee consisting of experts in health and social services who are appointed by a mayor.^63,64,65,66^ We applied the most severe care needs level for each targeted patient during each study period as an independent variable.

In addition, as a subanalysis, a joinpoint regression analysis was conducted to identify statistically significant changes in the trend of monthly medical expenditures in the 1 year before death using the National Cancer Institute’s Joinpoint trend analysis software.^67,68,69^ The extent of change in median unadjusted medical expenditures in each segment (period between 2 inflections) was estimated using the monthly percentage change. The number of segments was decided based on the algorithm’s best-fit recommendation.

The Jonckheere-Terpstra test and the analysis using generalized estimating equations were performed using SPSS for Windows, version 27.0 (IBM Corp). All statistical analyses were implemented with statistical significance set at 2-tailed *P* < .05.

## Results

According to the national health insurance claims for Nara Prefecture, the number of deaths among individuals aged 75 years or older between April 2014 and March 2018 was 34 317 (16 202 men [47.2%] and 18 115 women [52.8%]). Data for a total of 872 patients (2.5%) aged 100 to 104 years (131 men [15.0%] and 741 women [85.0%]) and 78 patients (0.2%) aged 105 to 109 years (fewer than 10 men) were extracted for data analysis. We could not report extracted data with counts of 9 or less because in Japan, national health insurance claims are administrative data, and a cell-size suppression policy is applied to them. This policy aims to avoid the risk that individuals will be identified. Based on the policy, supercentenarians were excluded as data analysis targets, although their presence was confirmed in the extracted data.^49^

The comparison between the extracted deaths and the official statistics, which are national public data, is shown in eTable 2 in the Supplement and indicated an overall match of 83.2%. Because the official statistics included deaths that occurred outside medical institutions and the claims data were missing information on certain patients (eg, persons receiving government welfare benefits because of economic hardship), the study’s data included most deaths that occurred while patients were receiving medical care.

The unadjusted medical expenditures and the proportion of inpatients among all patients in the last year of life by sex and age are shown in Table 1. In general, in the year before death, unadjusted median medical expenses were lower for women ($17 190 [IQR, $7294-$34 279]) than for men ($22 442 [IQR, $10 948-$40 533]). Although the median unadjusted outpatient expenditures among individuals aged 105 to 109 years were slightly higher than those among individuals aged 100 to 104 years, in other age groups, there was a trend of lower unadjusted medical expenditures in the last year of life among the older age groups.

**Table 1.** Unadjusted Medical Expenditures and the Proportion of Inpatients Among All Patients in the Last Year of Life by Sex and Age

Characteristic	Decedents, No. (%)	Median expenditures (IQR), US $^a^			Inpatients, No./total No. (%)
		Total	Hospitalization	Outpatient	
All	34 317 (100)	19 693 (8885-37 427)	17 216 (7212-34 306)	3430 (1817-6208)	30 185/34 317 (88.0)
Male	16 202 (47.2)	22 442 (10 948-40 533)	17 946 (7640-35 062)	3914 (2068-7079)	14 939/16 202 (92.2)
Female	18 115 (52.8)	17 190 (7294-34 279)	16 479 (6800-33 402)	3045 (1638-5422)	15 246/18 115 (84.2)
Noncentenarians, age, y					
75-79	4551 (13.3)	28 624 (14 265-48 420)	21 094 (9162-40 526)	4849 (2433-9410)	4311/4551 (94.7)
80-84	8076 (23.5)	24 273 (11 915-43 216)	19 096 (8233-37 547)	4090 (2168-7282)	7519/8076 (93.1)
85-89	9593 (28.0)	19 935 (9398-36 471)	17 444 (7094-33 703)	3395 (1842-5886)	8587/9593 (89.5)
90-94	7687 (22.4)	15 648 (7425-30 505)	15 029 (6455-30 479)	2899 (1568-5088)	6469/7687 (84.2)
95-99	3460 (10.1)	12 366 (5010-24 953)	13 457 (5614-26 927)	2633 (1469-4432)	2658/3460 (76.8)
Centenarians, age, y					
100-104	872 (2.5)	9399 (4003-19 458)	11 508 (4675-22 933)	2392 (1383-4596)	598/872 (68.6)
105-109	78 (0.2)	8321 (3003-20 483)	11 440 (5221-22 495)	2621 (1232-5131)	43/78 (55.1)

^a^ The IQR represents values at the 25th (quartile 1) and 75th (quartile 3) percentiles of medical expenditures.

The total median medical expenditures during the 1 year before death showed the same decreasing trend after calculating expenditures among all age groups for the total cohort and then separately by sex: the total median expenditures were $29 011 (IQR, $14 790-$48 724) for men aged 75 to 79 years and $8337 (IQR, $4027-$20 044) for men aged 105 to 109 years (*P* < .001), and the total median expenditures were $27 869 (IQR, $13 478-$47 464) for women aged 75 to 79 years and $8321 (IQR, $2851-$20 483) for women aged 105 to 109 years (*P* < .001) (Table 2 and Table 3).

**Table 2.** Unadjusted Medical Expenditures for Male Patients in the Last Year of Life by Age and the Proportion of Male Inpatients Among Total Male Patients

Age group	Decedents, No. (%)	Median expenditures (IQR), US $^a^			Inpatients, No./total No. (%)
		Total	Hospitalization	Outpatient	
All	16 202 (100)	22 442 (10 948-40 533)	17 946 (7640-35 062)	3914 (2068-7079)	14 939/16 202 (92.2)
Noncentenarians, age, y					
75-79	2956 (18.2)	29 011 (14 790-48 724)	20 302 (8998-39 906)	5068 (2500-9594)	2831/2956 (95.8)
80-84	4800 (30.0)^b^	25 418 (13 048-44 306)	19 261 (8427-37 418)	4331 (2326-7736)	(94.5)^b^
85-89	4765 (29.4)	20 866 (10 534-37 595)	17 557 (7182-33 497)	3679 (2019-6432)	4368/4765 (91.7)
90-94	2743 (16.9)	17 426 (8893-32 005)	14 891 (6539-30 385)	3247 (1735-5634)	2431/2743 (88.6)
95-99	797 (4.9)	14 161 (6990-27 547)	13 534 (6287-27 338)	3001 (1675-4842)	664/797 (83.3)
Centenarians, age, y					
100-104	131 (0.8)	14 835 (6475-29 597)	14 595 (6556-33 449)	2863 (1783-5473)	103/131 (78.6)
105-109	NR^c^	8337 (4027-20 044)	9335 (1390-28 861)	3947 (146-11 694)	(50.0)^c^

Abbreviation: NR, not reported.

^a^ The IQR represents values at the 25th (quartile 1) and 75th (quartile 3) percentiles of medical expenditures.

^b^ The number of male patients in the group aged 80 to 84 years was rounded up to prevent back-calculation of the number of male patients in the group aged 105 to 109 years. The percentage of male patients in the group aged 80 to 84 years was rounded up to prevent back-calculation of the number of male patients in this group.

^c^ Data extracted from national insurance claims with a count of 9 or fewer deaths among male patients in the group aged 105 to 109 years could not be reported owing to the cell-size suppression policy.

**Table 3.** Unadjusted Medical Expenditures for Female Patients in the Last Year of Life by Age and the Proportion of Female Inpatients Among Total Female Patients

Age group	Decedents, No. (%)	Median expenditures (IQR), US $^a^			Inpatients, No./total No. (%)
		Total	Hospitalization	Outpatient	
All	18 115 (100)	17 190 (7294-34 279)	16 479 (6800-33 402)	3045 (1638-5422)	15 246/18 115 (84.2)
Noncentenarians, age, y					
75-79	1595 (8.8)	27 869 (13 478-47 464)	22 183 (9671-41 755)	4354 (2215-8840)	1480/1595 (92.8)
80-84	3270 (18.0)^b^	22 346 (10 507-41 308)	18 744 (7847-37 947)	3733 (1963-6642)	(91.1)^b^
85-89	4828 (26.7)	18 906 (8401-35 190)	17 259 (6957-34 028)	3118 (1676-5294)	4219/4828 (87.4)
90-94	4944 (27.3)	14 682 (6647-29 571)	15 055 (6379-30 637)	2739 (1486-4775)	4038/4944 (81.7)
95-99	2663 (14.7)	11 570 (4630-23 856)	13 441 (5516-26 883)	2539 (1422-4289)	1994/2663 (74.9)
Centenarians, age, y					
100-104	741 (4.1)	8624 (3756-18 025)	10 586 (4554-21 718)	2308 (1348-4480)	495/741 (66.8)
105-109	NR^c^	8321 (2851-20 483)	11 440 (5380-22 495)	2619 (1234-5101)	(55.7)^c^

Abbreviation: NR, not reported.

^a^ The IQR represents values at the 25th (quartile 1) and 75th (quartile 3) percentiles of medical expenditures.

^b^ The number of female patients in the group aged 80 to 84 years was rounded up to prevent back-calculation of the number of female patients in the group aged 105 to 109 years. The percentage of female patients in the group aged 80 to 84 years was rounded up to prevent back-calculation of the number of female patients in this group.

^c^ The number of female patients in the group aged 105 to 109 years was more than 9; however, to prevent back-calculation of the small number of male patients in this group, it is not reported.

The proportions of inpatients among all patients and by sex and age group during the 1 year before death also decreased in association with increasing age: 4311 of all 4551 patients aged 75 to 79 years (94.7%); 43 of all 78 patients aged 105 to 109 years (55.1%); 2831 of 2956 men aged 75 to 79 years (95.8%); 50.0% of men aged 105 to 109 years (the number is not reported owing to the small sample size); 1480 of 1595 women aged 75 to 79 years (92.8%); and 55.7% of women aged 105 to 109 years (the number is not reported to prevent back-calculation of the number of men) (Table 1, Table 2, and Table 3). Specifically, 274 of 872 younger centenarians (31.4%) and 35 of 78 semi-supercentenarians (44.9%) had not been admitted to a hospital in the year before death.

The mean unadjusted per-patient medical expenditures for the 1 year before death by sex and age are shown in eTables 3 to 5 in the Supplement. Although the mean values were generally higher than the median values because of outlier effects, mean unadjusted medical expenditures in the year before death tended to be lower in older age groups, as did the trends of median values.

**Figure 1.**
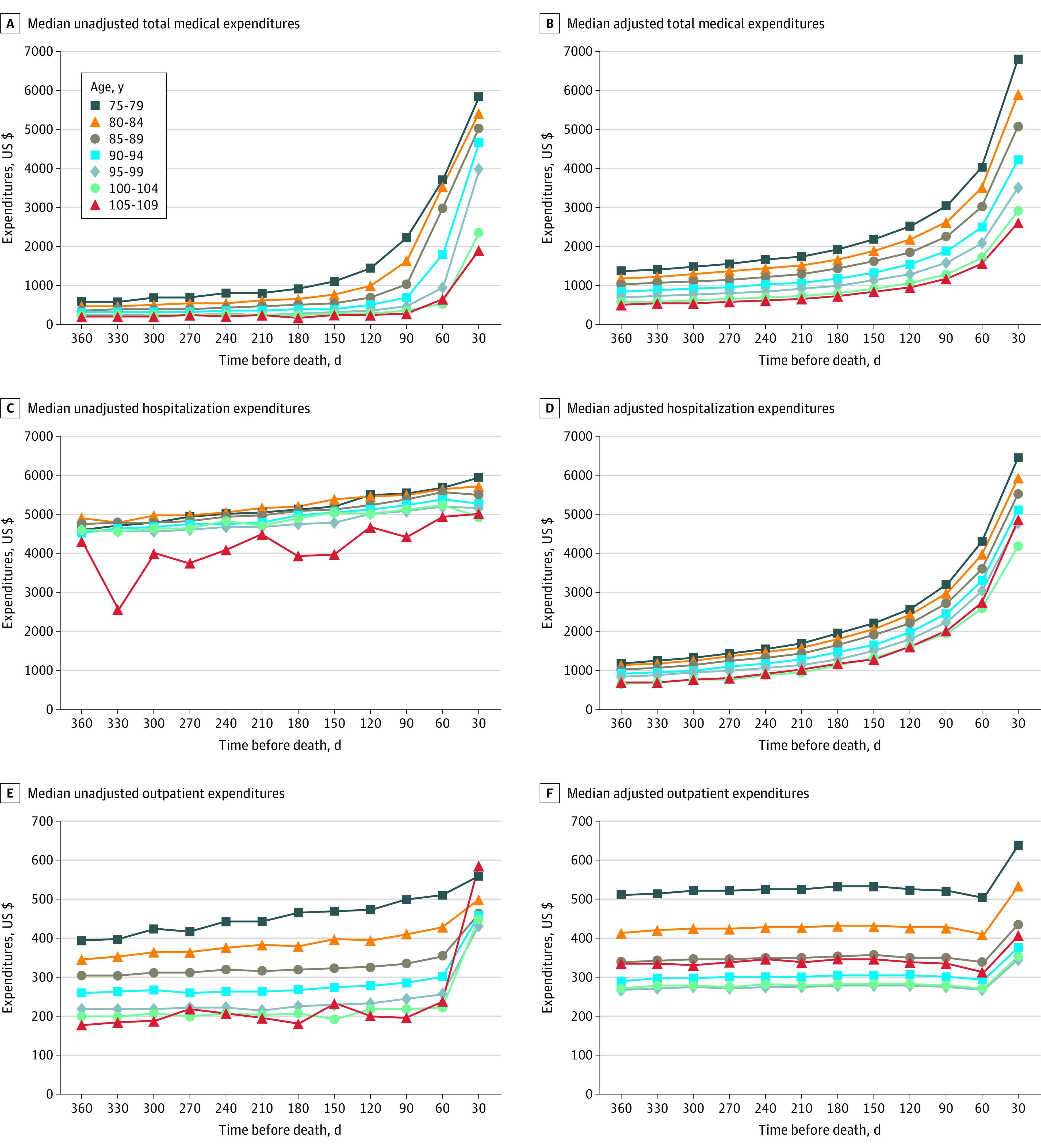
Median Unadjusted and Adjusted Medical Expenditures by Age Group in the Year Before Death The calculations targeted unique patients who incurred expenditures during each 30-day period in the year before death.

**Figure 2.**
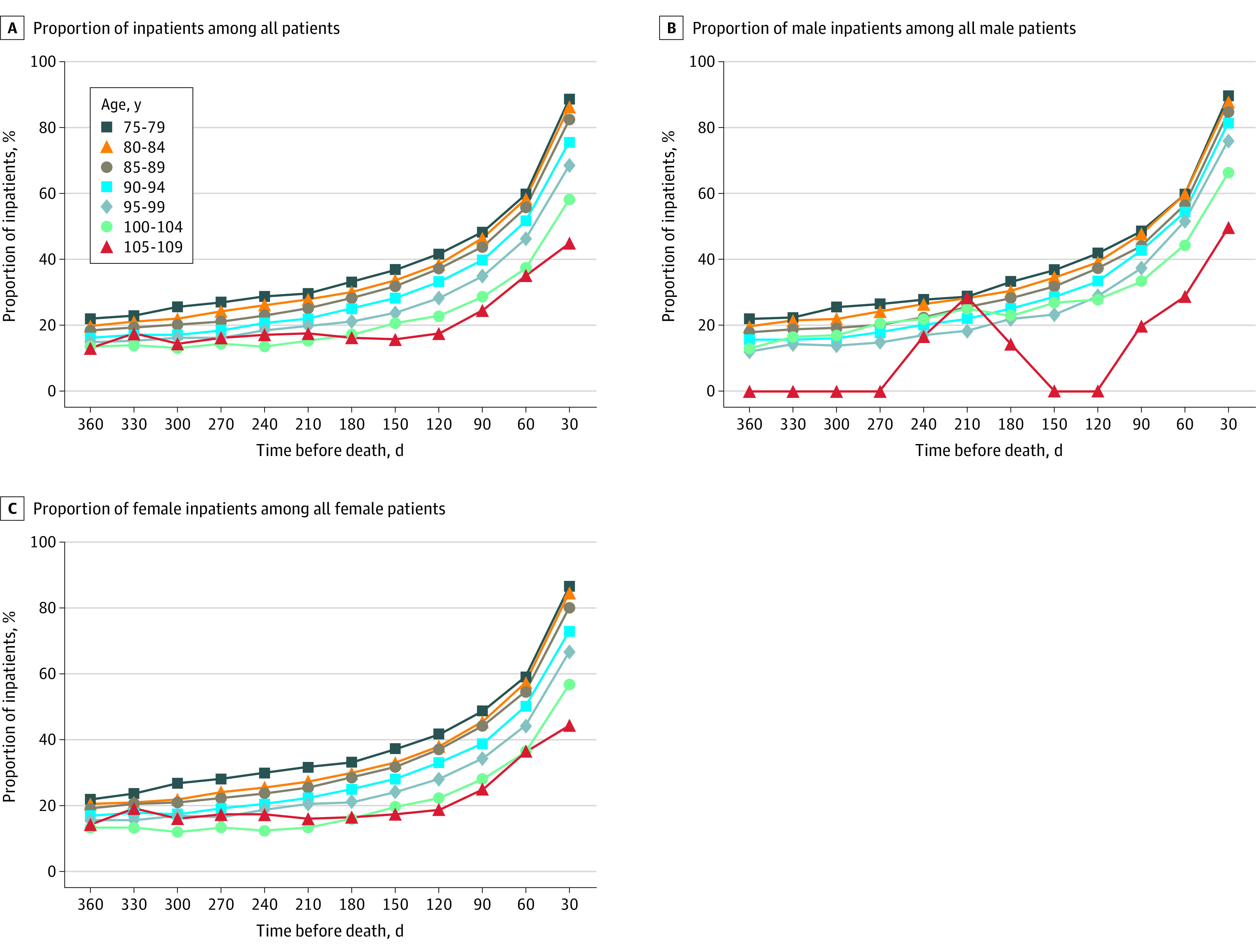
Proportion of Inpatients Among All Patients by Sex and Age Group in the Year Before Death The proportion of inpatients among all patients during every 30-day period in the year before death was calculated by dividing the number of patients who incurred hospitalization expenditures by the number of unique patients who incurred hospitalization and/or outpatient expenditures.

The median adjusted total expenditures during the 30 days before death were $6784 (IQR, $4884-$9703) for ages 75 to 79 years, $5894 (IQR, $4292-$8536) for 80 to 84 years, $5069 (IQR, $3676-$7150) for 85 to 89 years, $4205 (IQR, $3085-$5914) for 90 to 94 years, $3522 (IQR, $2626-$4861) for 95 to 99 years, $2898 (IQR, $2241-$3835) for 100 to 104 years, and $2626 (IQR, $1938-$3527) for 105 to 109 years. The median medical expenditures by age group for every 30-day period in the year before death are shown in Figure 1. The median unadjusted and adjusted total medical expenditures for each 30-day period decreased in association with increasing age; residents aged 105 to 109 years had the lowest median expenditures (unadjusted, $1945 [IQR, $686-$4976]; adjusted, $2626 [IQR, $1938-$3527]), and those aged 75 to 79 years had the highest median expenditures (unadjusted, $5862 [IQR, $3414-$8082]; adjusted, $6784 [IQR, $4884-$9703]) (Figure 1A and B). The distribution of every independent variable in the generalized estimating equations analysis for total medical expenditures is shown in eTable 6 in the Supplement. Residents aged 105 to 109 years had the second-lowest median unadjusted hospitalization expenditures ($5012 [IQR, $2830-$6427]) in the 30 days before death, and those aged 100 to 104 years had the lowest ($4950 [IQR, $2923-$6175]). However, residents aged 105 to 109 years had slightly higher median adjusted hospitalization expenditures during the 30 days before death ($4871 [IQR, $3661-$5841]) compared with those aged 95 to 99 years ($4772 [IQR, $3486-$6471]) (Figure 1C and D). The trends in outpatient expenditures in the 30 days before death were different from those of total and hospitalization expenditures. The median unadjusted outpatient expenditures for individuals were highest for individuals aged 105 to 109 years ($585 [IQR, $247-$1462]) and second highest for those aged 75 to 79 years ($558 [IQR, $242-$1382]). The median adjusted outpatient expenditures were the fourth highest for individuals aged 105 to 109 years ($406 [IQR, $316-$519]) and highest for those aged 75 to 79 years ($640 [IQR, $427-$931]) (Figure 1E and F). The proportion of inpatients among all patients by sex and age group every 30 days in the year before death is shown in Figure 2. The proportion of inpatients during the 30 days before death decreased in association with increasing age: 4041 of all 4551 patients aged 75 to 79 years (88.8%); 35 of all 78 patients aged 105 to 109 years (44.9%); 2660 of 2956 men aged 75 to 79 years (90.0%); 50.0% of men aged 105 to 109 years (the number is not reported owing to the small sample size); 1381 of 1595 women aged 75 to 79 years (86.6%); and 44.3% of women aged 105 to 109 years (the number is not reported to prevent back-calculation of the number of men) (Figure 2).

In the subanalysis of the joinpoint regression, the monthly percentage change values in median unadjusted total expenditures at segment 1 (3 or 4 months before death) showed a statistically significant decrease in all age groups. Furthermore, the mean monthly percentage change values decreased significantly with similar magnitude among all age groups (eTable 7 in the Supplement). In terms of hospitalization expenditures, there were no significant differences in monthly percentage change and mean monthly percentage change values in the groups aged 100 to 104 years and 105 to 109 years (eTable 8 in the Supplement). The monthly percentage change values of outpatient expenditures at segment 1 (3 months before death) and the mean monthly percentage change values decreased significantly in all age groups excluding the monthly percentage change value in the group aged 75 to 79 years. The decreases were larger in the older age groups (eTable 9 in the Supplement).

## Discussion

This cohort study used large-scale insurance claims data to identify and compare the medical expenditures of centenarians and noncentenarians aged 75 years or older in Japan in the year before their death. Medical expenditures tended to be lower in the older age groups. Semi-supercentenarians (aged 105 to 109 years) had the lowest median total expenditures in the year before death, whereas their outpatient care costs tended to be higher. The proportion of inpatients among all patients in the last year of life also decreased in the older age groups. Of interest, 31.4% of the younger centenarians and 44.9% of the semi-supercentenarians were not admitted to a hospital in the year before death. The comparison of median premortem medical expenditures of different age groups in each 30-day period revealed that in almost all periods, the median total and hospitalization expenditures for the centenarian group were lower than those for the noncentenarian group. However, the median outpatient expenditures for the centenarian group were not lower than those for the noncentenarian group, indicating trends different from those found for total and hospitalization expenditures.

Previous studies have indicated that premortem medical expenditures for older individuals tended to decrease as age increased; this finding has been shown in different countries with various health care systems, including Japan.^32,33,34,35,36,37,38,39,40,41,42,43,44,45,46^ These prior studies primarily used the mean values of medical expenditures per patient.^32,33,40,41,43,44^ However, based on previous findings that medical expenditures may not be normally distributed and that the mean may be affected by outliers,^70,71,72^ the current study used median values for medical expenditures per patient as primary outcome data. Despite using median values, the results showed trends similar to those found in previous studies^32,33,40,41,43,44^; premortem medical expenditures tended to decrease as patient age increased.

To our knowledge, this is the first study to distinguish between the medical expenditures incurred monthly during the 1 year before death for younger centenarians and semi-supercentenarians by sex and age group; previous studies of centenarians^43,44,45,46^ did not include these variables. Hazra et al^43^ used electronic health records between 2010 and 2014 in the UK to analyze centenarians’ mean annual predicted health care costs in the last year of life, but they targeted individuals aged up to 105 years and did not distinguish the predicted costs between sexes. In the present study, women’s medical expenditures tended to be lower in all age groups. The extracted insurance claims data on the number of deaths showed that women outnumbered men in the older age groups. To examine whether the lower medical expenditures in the older age groups were associated with the presence of more women, we analyzed the premortem medical expenditure trends by sex and age group. The results showed that premortem medical expenditures tended to decrease in association with increasing age for both men and women.

A previous 29-year follow-up cohort study of Danish centenarians and noncentenarians^16^ reported that centenarians had been healthier than their contemporaries who died at younger ages. Within a longitudinal framework, the study found a clear and consistent inverse association between being hospitalized and the length of stay in a hospital and age at death.^16^ A recent cohort study^12^ using health insurance data on German centenarians, nonagenarians (aged 90 to 99 years), and octogenarians (aged 80 to 89 years) showed that individuals who died as centenarians had comorbidity trends during the 6 years before death that were different from those of individuals in the other 2 age groups. Centenarians had both fewer comorbidities and a lesser increase in the number of comorbidities as they approached death.^12^ Another cohort study^18^ focusing on Japanese centenarians and supercentenarians reported that the mean Barthel index scores at various ages of entry across the 3 age groups (ie, supercentenarians, semi-supercentenarians, and younger centenarians) showed significantly higher physical functioning for supercentenarians compared with the other 2 age groups at any age. Supercentenarians had unique traits: the postponement of functional decline and maintenance of physical independence until very late in life.^18^ The centenarians included in the current study were aged up to 109 years, but the results showing decreasing medical expenditures and proportions of inpatients among all patients in older age groups are consistent with those of previous clinical studies on centenarians.^12,16,18^ The finding that less variation was found in medical expenditures per patient among centenarians than among noncentenarians is also consistent with the findings of previous clinical studies.^12,16,18^

This study examined complete 5-year health and LTC insurance claims data on health care services for patients aged 75 years or older in Nara Prefecture. Because Japan has the world’s highest proportion of centenarians among the general population,^4^ this study’s novel results may be generalizable and useful for assessing premortem medical expenditure trends for centenarians in other countries. Because the association between centenarians’ health care services coverage and medical costs is still unclear, it is imperative to study centenarians’ outpatient care, including home medical care; in this study, 44.9% of semi-supercentenarians were not hospitalized during the year before death and the highest median unadjusted outpatient expenditures were incurred in the 30 days before death.

In our analysis, we did not analyze centenarians’ premortem medical expenditures according to the presence of conditions such as hypertension, type 2 diabetes, dementia, cancer, heart disease, and stroke or their accompanying medical treatments because the primary aim was to explore how age is associated with medical expenditures. However, it is important to emphasize that centenarians have differences in health status in terms of independence level (as measured by the Barthel index) and the presence of chronic illnesses. In addition, the trajectory of differences between centenarians and noncentenarians with respect to disability in the last year of life remains unknown.^73^ As such, we suggest that future studies consider in greater detail the factors associated with centenarians’ premortem periods of serious illness.

### Limitations

This study has limitations. First, the insurance claims data did not include information on cause of death; thus, our analysis did not consider cause of death. Second, our analysis focused on the prefectural-level population, which resulted in fewer than 10 supercentenarians extracted from the insurance claims data. Consequently, supercentenarians were excluded from the analysis based on the cell-size suppression policy.

## Conclusions

Using large-scale insurance claims data, this cohort study found that Japanese centenarians’ medical expenditures in the last year of life were generally lower than those of noncentenarians aged 75 years or older. The proportion of centenarians, especially semi-supercentenarians, who were not admitted to a hospital in the year before death was higher than that of noncentenarians, whereas the median unadjusted outpatient care costs were the highest for semi-supercentenarians in the 30 days before death. Centenarians’ characteristic shorter periods of serious illness compared with other age groups, as reported by previous clinical studies,^12,13,14,15,16,17,18^ was observed through health care services coverage as lower premortem medical expenditures and lower proportions of inpatients. However, research on public health policy for centenarians is scarce. Further detailed investigation of centenarians’ health care services coverage, including home medical care, linked to medical expenditures appears to be needed to inform future public health policy for an increasing population of centenarians.

## Notes

Supplement. **eTable 1.** Top 200 Most Frequent Diagnosis-Related Codes Without Suspected Disease Flag and Disease Names of *ICD-10* 3-Character Code Block Categories Among Targeted Decedents for the Generalized Estimating Equations Analysis **eTable 2.** Number of Deaths from Nara Prefecture Claims Data and Official Statistics **eTable 3.** Average Unadjusted Medical Expenditures Per Patient in the Last Year of Life by Sex and Age of All Patients **eTable 4.** Average Unadjusted Medical Expenditures Per Patient in the Last Year of Life by Age: Male Patients **eTable 5. **Average Unadjusted Medical Expenditures Per Patient in the Last Year of Life by Age: Female Patients **eTable 6. **Distribution of Every Independent Variable in the Generalized Estimating Equations Analysis for Total Medical Expenditures **eTable 7. **Joinpoint Regression Results for Median Unadjusted Total Medical Expenditures for 1 Year Prior to Death by Age Group and Month **eTable 8. **Joinpoint Regression Results for Median Unadjusted Hospitalization Expenditures for 1 Year Prior to Death by Age Group and Month **eTable 9. **Joinpoint Regression Results for Median Unadjusted Outpatient Expenditures for 1 Year Prior to Death by Age Group and Month
