# Supplementary material for: Comparison of Japanese Centenarians’ and Noncentenarians’ Medical Expenditures in the Last Year of Life
Source: JAMA Netw Open. 2021 Nov 5;4(11):e2131884. doi: 10.1001/jamanetworkopen.2021.31884 (PMC8571656; doi:10.1001/jamanetworkopen.2021.31884)
Supplement: Supplement. — eTable 1. Top 200 Most Frequent Diagnosis-Related Codes Without Suspected Disease Flag and Disease Names of ICD-10 3-Character Code Block Categories Among Targeted Decedents for the Generalized Estimating Equations Analysis eTable 2. Number of Deaths from Nara Prefecture Claims Data and Official Statistics eTable 3. Average Unadjusted Medical Expenditures Per Patient in the Last Year of Life by Sex and Age of All Patients eTable 4. Average Unadjusted Medical Expenditures Per Patient in the Last Year of Life by Age: Male Patients eTable 5. Average Unadjusted Medical Expenditures Per Patient in the Last Year of Life by Age: Female Patients eTable 6. Distribution of Every Independent Variable in the Generalized Estimating Equations Analysis for Total Medical Expenditures eTable 7. Joinpoint Regression Results for Median Unadjusted Total Medical Expenditures for 1 Year Prior to Death by Age Group and Month eTable 8. Joinpoint Regression Results for Median Unadjusted Hospitalization Expenditures for 1 Year Prior to Death by Age Group and Month eTable 9. Joinpoint Regression Results for Median Unadjusted Outpatient Expenditures for 1 Year Prior to Death by Age Group and Month [file jamanetwopen-e2131884-s001.pdf]

Supplementary Online Content

Nakanishi Y, Tsugihashi Y, Akahane M, et al. Comparison of Japanese centenarians' and noncentenarians' medical expenditures in the last year of life. *JAMA Netw Open*. 2021;4(11):e2131884. doi:10.1001/jamanetworkopen.2021.31884

eTable 1. Top 200 Most Frequent Diagnosis-Related Codes Without Suspected Disease Flag and Disease Names of *ICD-10* 3-Character Code Block Categories Among Targeted Decedents for the Generalized Estimating Equations Analysis

eTable 2. Number of Deaths from Nara Prefecture Claims Data and Official Statistics

eTable 3. Average Unadjusted Medical Expenditures Per Patient in the Last Year of Life by Sex and Age of All Patients

eTable 4. Average Unadjusted Medical Expenditures Per Patient in the Last Year of Life by Age: Male Patients

eTable 5. Average Unadjusted Medical Expenditures Per Patient in the Last Year of Life by Age: Female Patients

eTable 6. Distribution of Every Independent Variable in the Generalized Estimating Equations Analysis for Total Medical Expenditures

eTable 7. Joinpoint Regression Results for Median Unadjusted Total Medical Expenditures for 1 Year Prior to Death by Age Group and Month

eTable 8. Joinpoint Regression Results for Median Unadjusted Hospitalization Expenditures for 1 Year Prior to Death by Age Group and Month

eTable 9. Joinpoint Regression Results for Median Unadjusted Outpatient Expenditures for 1 Year Prior to Death by Age Group and Month

This supplementary material has been provided by the authors to give readers additional information about their work.

© 2021 Nakanishi Y et al. *JAMA Network Open*.

eTable 1. Top 200 Most Frequent Diagnosis-Related Codes without Suspected Disease Flag and Disease Names of *ICD-10* 3-Character Code Block Categories Among Targeted Decedents for the Generalized Estimating Equations Analysis

ICD-10 code (descending order of frequency)	Disease name
K59	Other functional intestinal disorders
I10	Essential (primary) hypertension
I50	Heart failure
K29	Gastritis and duodenitis
K21	Gastro-esophageal reflux disease
L30	Other dermatitis
G47	Sleep disorders
M54	Dorsalgia
E86	Volume depletion
J18	Pneumonia, organism unspecified
K25	Gastric ulcer
L85	Other epidermal thickening
J96	Respiratory failure, not elsewhere classified
E78	Disorders of lipoprotein metabolism and other lipidemias
H52	Disorders of refraction and accommodation
H10	Conjunctivitis
J20	Acute bronchitis
I20	Angina pectoris
E14	Unspecified diabetes mellitus
M47	Spondylosis
T14	Injury of unspecified body region
J06	Acute upper respiratory infections of multiple and unspecified sites

© 2021 Nakanishi Y et al. *JAMA Network Open*.

M81	Osteoporosis without pathological fracture
B35	Dermatophytosis
I63	Cerebral infarction
M62	Diastasis of muscle
D50	Iron deficiency anemia
A09	Other gastroenteritis and colitis of infectious and unspecified origin
M17	Gonarthrosis [arthrosis of knee]
L89	Decubitus ulcer and pressure area
I69	Sequelae of cerebrovascular disease
J40	Bronchitis, not specified as acute or chronic
R63	Symptoms and signs concerning food and fluid intake
G30	Alzheimer disease
E87	Other disorders of fluid, electrolyte and acid-base balance
J69	Pneumonitis due to solids and liquids
H26	Other cataract
F00	Dementia in Alzheimer disease (G30.-†)
N39	Other disorders of urinary system
J30	Vasomotor and allergic rhinitis
J42	Unspecified chronic bronchitis
G62	Other polyneuropathies
J45	Asthma
F03	Unspecified dementia
K76	Other diseases of liver
N30	Cystitis
D64	Other anemias
R13	Dysphagia
R09	Other symptoms and signs involving the circulatory and respiratory systems
I49	Other cardiac arrhythmias
E11	Non-insulin-dependent diabetes mellitus
R52	Pain, not elsewhere classified

© 2021 Nakanishi Y et al. *JAMA Network Open*.

N40	Hyperplasia of prostate
I70	Atherosclerosis
J02	Acute pharyngitis
H16	Keratitis
Z96	Presence of other functional implants
I48	Atrial fibrillation and flutter
R60	Edema, not elsewhere classified
J90	Pleural effusion, not elsewhere classified
R11	Nausea and vomiting
E79	Disorders of purine and pyrimidine metabolism
L98	Other disorders of skin and subcutaneous tissue, not elsewhere classified
M48	Other spondylopathies
M75	Shoulder lesions
R57	Shock, not elsewhere classified
M79	Other soft tissue disorders, not elsewhere classified
E88	Other metabolic disorders
L29	Pruritus
N18	Chronic kidney disease
F20	Schizophrenia
F32	Depressive episode
H04	Disorders of lacrimal system
K92	Other diseases of digestive system
R42	Dizziness and giddiness
N31	Neuromuscular dysfunction of bladder, not elsewhere classified
K12	Stomatitis and related lesions
N32	Other disorders of bladder
J00	Acute nasopharyngitis [common cold]
H40	Glaucoma
R40	Somnolence, stupor and coma
N19	Unspecified kidney failure

© 2021 Nakanishi Y et al. *JAMA Network Open*.

S00	Superficial injury of head
K80	Cholelithiasis
E03	Other hypothyroidism
E56	Other vitamin deficiencies
K64	Hemorrhoids and perianal venous thrombosis
F41	Other anxiety disorders
H35	Other retinal disorders
S32	Fracture of lumbar spine and pelvis
S72	Fracture of femur
R33	Retention of urine
N28	Other disorders of kidney and ureter, not elsewhere classified
M13	Other arthritis
A41	Other sepsis
L03	Cellulitis
R10	Abdominal and pelvic pain
R50	Fever of other and unknown origin
H25	Senile cataract
M25	Other joint disorders, not elsewhere classified
A49	Bacterial infection of unspecified site
J15	Bacterial pneumonia, not elsewhere classified
E63	Other nutritional deficiencies
I46	Cardiac arrest
C78	Secondary malignant neoplasm of respiratory and digestive organs
J43	Emphysema
S22	Fracture of rib(s), sternum and thoracic spine
H01	Other inflammation of eyelid
U82	Resistance to betalactam antibiotics
I25	Chronic ischemic heart disease
L25	Unspecified contact dermatitis
F48	Other neurotic disorders

© 2021 Nakanishi Y et al. *JAMA Network Open*.

M19	Other arthrosis
C16	Malignant neoplasm of stomach
G40	Epilepsy
H61	Other disorders of external ear
J44	Other chronic obstructive pulmonary disease
D63	Anemia in chronic diseases classified elsewhere
R31	Unspecified hematuria
L08	Other local infections of skin and subcutaneous tissue
R51	Headache
G20	Parkinson disease
M53	Other dorsopathies, not elsewhere classified
H02	Other disorders of eyelid
R00	Abnormalities of heart beat
H53	Visual disturbances
E46	Unspecified protein-energy malnutrition
H36	Retinal disorders in diseases classified elsewhere
C34	Malignant neoplasm of bronchus and lung
F05	Delirium, not induced by alcohol and other psychoactive substances
K56	Paralytic ileus and intestinal obstruction without hernia
K63	Other diseases of intestine
R25	Abnormal involuntary movements
B02	Zoster [herpes zoster]
K73	Chronic hepatitis, not elsewhere classified
R26	Abnormalities of gait and mobility
I67	Other cerebrovascular diseases
L50	Urticaria
B18	Chronic viral hepatitis
I74	Arterial embolism and thrombosis
H60	Otitis externa
H90	Conductive and sensorineural hearing loss

© 2021 Nakanishi Y et al. *JAMA Network Open*.

J32	Chronic sinusitis
R39	Other symptoms and signs involving the urinary system
J84	Other interstitial pulmonary diseases
L27	Dermatitis due to substances taken internally
F50	Eating disorders
I65	Occlusion and stenosis of precerebral arteries, not resulting in cerebral infarction
I80	Phlebitis and thrombophlebitis
K58	Irritable bowel syndrome
E53	Deficiency of other B group vitamins
C18	Malignant neoplasm of colon
D69	Purpura and other hemorrhagic conditions
I35	Nonrheumatic aortic valve disorders
S30	Superficial injury of abdomen, lower back and pelvis
I21	Acute myocardial infarction
I73	Other peripheral vascular diseases
I51	Complications and ill-defined descriptions of heart disease
K83	Other diseases of biliary tract
J10	Influenza due to other identified influenza virus
G64	Other disorders of peripheral nervous system
K52	Other noninfective gastroenteritis and colitis
Z95	Presence of cardiac and vascular implants and grafts
T81	Complications of procedures, not elsewhere classified
E83	Disorders of mineral metabolism
D65	Disseminated intravascular coagulation [defibrination syndrome]
K31	Other diseases of stomach and duodenum
Z93	Artificial opening status
E77	Disorders of glycoprotein metabolism
S01	Open wound of head
I71	Aortic aneurysm and dissection
C79	Secondary malignant neoplasm of other and unspecified sites

© 2021 Nakanishi Y et al. *JAMA Network Open*.

N12	Tubulo-interstitial nephritis, not specified as acute or chronic
B37	Candidiasis
M16	Coxarthrosis [arthrosis of hip]
H81	Disorders of vestibular function
N17	Acute renal failure
L84	Corns and callosities
A04	Other bacterial intestinal infections
C61	Malignant neoplasm of prostate
M06	Other rheumatoid arthritis
R07	Pain in throat and chest
F45	Somatoform disorders
S09	Other and unspecified injuries of head
M10	Gout
I95	Hypotension
S06	Intracranial injury
I34	Nonrheumatic mitral valve disorders
R19	Other symptoms and signs involving the digestive system and abdomen
R04	Hemorrhage from respiratory passages
D48	Neoplasm of uncertain or unknown behavior of other and unspecified sites
J11	Influenza, virus not identified
K86	Other diseases of pancreas
B00	Herpesviral [herpes simplex] infections
R06	Abnormalities of breathing
K74	Fibrosis and cirrhosis of liver
K85	Idiopathic acute pancreatitis
N20	Calculus of kidney and ureter
R18	Ascites
G46	Vascular syndromes of brain in cerebrovascular diseases (I60-I67+)

© 2021 Nakanishi Y et al. *JAMA Network Open*.

eTable 2. Number of Deaths from Nara Prefecture Claims Data and Official Statistics

	Claims data in Nara Prefecture (Nara)			Official statistics (MHLW)			Comparison		
**Period**	**Apr. 2014–Mar. 2018 (48 months)**			**Jan. 2014–Dec. 2017 (48 months)**			**Nara/MHLW**		
**Age group**	**Number of deaths (n)**			**Number of deaths (n)**			**Percentages (%)**		
	All	Male	Female	All	Male	Female	All	Male	Female
All	34,317	16,202	18,115	41,227	18,737	22,490	83.2	86.5	80.5
Noncentenarian group									
75–79[a]	4,551	2,956	1,595	5,593	3,538	2,055	81.4	83.6	77.6
80–84	8,076	4,800[d]	3,270[d]	9,753	5,631	4,122	82.8	85.0[e]	79.0[e]
85–89	9,593	4,765	4,828	11,431	5,468	5,963	83.9	87.1	81.0
90–94	7,687	2,743	4,944	9,058	3,063	5,995	84.9	89.6	82.5
95–99	3,460	797	2,663	4,171	876	3,295	83.0	91.0	80.8
Centenarian group									
100–104	872	131	741	1,128	153	975	77.3	85.6	76.0
105–109	78	-[b]	-[c]	93	8	85	83.9	-	-

Note: Official statistics (MHLW) include deaths outside of a medical institution. Insurance claims data in Nara Prefecture do not contain data on certain patients, such as those receiving government welfare benefits because of economic hardship. MHLW=Ministry of Health, Labour and Welfare of Japan.

[a] The number of deaths in the 75–79 years age group did not include decedents who died at 75 years. Thus, Nara Prefecture claims data on decedents who died at 76–79 years was used in the comparison with the official statistics for the 76–79 years age group.

[b] Extracted data from national insurance claims with count ≤ 9 (number of deaths in the 105–109 years age group by sex) cannot be reported due to the cell size suppression policy.

[c] The number of women in the 105–109 years age group is greater than nine; however, the number of women in this group cannot be reported to prevent back-calculation of the number of men in the age group.

[d] The numbers of men and women in the 80–84 years age group are rounded up to prevent back-calculation of the number of men in the 105–109 years age group.

[e] The percentages of extracted numbers of male and female deaths in the 80–84 years age group to those from official statistics are rounded up to prevent back-calculation of the numbers of male and female deaths in the age group.

© 2021 Nakanishi Y et al. *JAMA Network Open*.

eTable 3. Average Unadjusted Medical Expenditures Per Patient in the Last Year of Life by Sex and Age of All Patients

Characteristic	Decedents, n (%)	Total expenditures	Hospitalization expenditures	Outpatient expenditures
		Mean (SD), $	Mean (SD), $	Mean (SD), $
All	34,317	26,094 (22,631)	23,893 (22,190)	5,431 (7,190)
Male	16,202 (47.2)	28,624 (23,389)	24,619 (22,736)	6,248 (8,140)
Female	18,115 (52.8)	23,831 (21,684)	23,182 (21,618)	4,680 (6,095)
Age group				
Noncentenarian group				
75–79	4,551 (13.3)	34,503 (26,789)	28,207 (25,355)	8,263 (10,590)
80–84	8,076 (23.5)	30,492 (24,484)	26,180 (23,951)	6,527 (8,193)
85–89	9,593 (28.0)	25,826 (21,403)	23,570 (21,492)	5,078 (6,546)
90–94	7,687 (22.4)	21,721 (19,075)	21,232 (19,485)	4,116 (4,751)
95–99	3,460 (10.1)	18,273 (17,740)	19,513 (18,598)	3,534 (3,683)
Centenarian group				
100–104	872 (2.5)	15,165 (15,944)	17,401 (17,508)	3,433 (3,174)
105–109	78 (0.2)	13,079 (13,900)	17,031 (15,986)	3,998 (4,255)

Abbreviation: SD, standard deviation.

© 2021 Nakanishi Y et al. *JAMA Network Open*.

eTable 4. Average Unadjusted Medical Expenditures Per Patient in the Last Year of Life by Age: Male Patients

Age group	Decedents, n (%)	Total expenditures	Hospitalization expenditures	Outpatient expenditures
		Mean (SD), $	Mean (SD), $	Mean (SD), $
All	16,202	28,624 (23,389)	24,619 (22,736)	6,248 (8,140)
Noncentenarian group				
75–79	2,956 (18.2)	34,846 (26,846)	27,959 (25,510)	8,501 (10,964)
80–84	4,800[b] (30.0[c])	31,503 (24,554)	26,358 (23,985)	6,974 (8,652)
85–89	4,765 (29.4)	26,866 (21,664)	23,623 (21,631)	5,515 (6,923)
90–94	2,743 (16.9)	23,115 (19,228)	21,070 (19,304)	4,641 (5,592)
95–99	797 (4.9)	19,268 (16,403)	18,704 (16,505)	3,870 (3,770)
Centenarian group				
100–104	131 (0.8)	19,942 (17,462)	20,716 (18,317)	3,860 (2,941)
105–109	-[a] (-)	12,062 (11,052)	13,195 (14,895)	6,246 (7,811)

Abbreviation: SD, standard deviation.

[a] Extracted data from national insurance claims with count ≤ 9 (number of deaths in male patients in the 105–109 years age group) cannot be reported due to the cell size suppression policy.

[b] The number of male patients in the 80–84 years age group is rounded up to prevent back-calculation of the number of male patients in the 105–109 years age group.

[c] The percentage of male patients in the 80–84 years age group is rounded up to prevent back-calculation of the number of male patients in the age group.

© 2021 Nakanishi Y et al. *JAMA Network Open*.

eTable 5. Average Unadjusted Medical Expenditures Per Patient in the Last Year of Life by Age: Female Patients

Age group	Decedents, n (%)	Total expenditures	Hospitalization expenditures	Outpatient expenditures
		Mean (SD), $	Mean (SD), $	Mean (SD), $
All	18,115	23,831 (21,684)	23,182 (21,618)	4,680 (6,095)
Noncentenarian group				
75–79	1,595 (8.8)	33,867 (26,679)	28,682 (25,057)	7,811 (9,828)
80–84	3,270[b] (18.0[c])	29,009 (24,308)	25,908 (23,900)	5,858 (7,404)
85–89	4,828 (26.7)	24,801 (21,094)	23,516 (21,349)	4,635 (6,108)
90–94	4,944 (27.3)	20,948 (18,948)	21,330 (19,595)	3,814 (4,163)
95–99	2,663 (14.7)	17,975 (18,114)	19,783 (19,241)	3,430 (3,650)
Centenarian group				
100–104	741 (4.1)	14,321 (15,520)	16,711 (17,274)	3,357 (3,210)
105–109	-[a] (-)	13,196 (14,250)	17,424 (16,224)	3,756 (3,709)

Abbreviation: SD, standard deviation.

[a] The number of female patients in the 105–109 years age group is over nine; however, it cannot be reported to prevent back-calculation of the number of male patients in the 105–109 age group.

[b] The number of female patients in the 80–84 years age group is rounded up to prevent back-calculation of the number of female patients in the 105–109 years age group.

[c] The percentage of female patients in the 80–84 years age group is rounded up to prevent back-calculation of the number of female patients in this group.

© 2021 Nakanishi Y et al. *JAMA Network Open*.

eTable 6. Distribution of Every Independent Variable in the Generalized Estimating Equations Analysis for Total Medical Expenditures

Independent variable	Number of corresponded targeted patients
Sex	
Male	16,202
Female	18,119
Age group	
75–79	4,551
80–84	8,076
85–89	9,593
90–94	7,687
95–99	3,460
100–104	872
105–109	78
Care-needs levels	
No certification	5,459
Support level 1	827
Support level 2	1,312
Care-needs level 1	2,256
Care-needs level 2	3,958
Care-needs level 3	4,881
Care-needs level 4	7,359
Care-needs level 5	8,269
Diagnosis-related code (disease name of ICD-10 three-character code block categories)	
K59 (Other functional intestinal disorders)	29,351
I10 (Essential (primary) hypertension)	27,104
I50 (Heart failure)	22,475
K29 (Gastritis and duodenitis)	21,683

© 2021 Nakanishi Y et al. *JAMA Network Open*.

K21 (Gastro-esophageal reflux disease)	19,277
L30 (Other dermatitis)	18,984
G47 (Sleep disorders)	17,954
M54 (Dorsalgia)	17,712
E86 (Volume depletion)	17,515
J18 (Pneumonia, organism unspecified)	17,061
K25 (Gastric ulcer)	15,069
L85 (Other epidermal thickening)	14,646
J96 (Respiratory failure, not elsewhere classified)	14,409
E78 (Disorders of lipoprotein metabolism and other lipidemias)	14,161
H52 (Disorders of refraction and accommodation)	13,883
H10 (Conjunctivitis)	13,222
J20 (Acute bronchitis)	13,140
I20 (Angina pectoris)	13,094
E14 (Unspecified diabetes mellitus)	13,045
M47 (Spondylosis)	12,741
T14 (Injury of unspecified body region)	12,667
J06 (Acute upper respiratory infections of multiple and unspecified sites)	12,453
M81 (Osteoporosis without pathological fracture)	12,273
B35 (Dermatophytosis)	11,923
I63 (Cerebral infarction)	11,757
M62 (Diastasis of muscle)	11,561
D50 (Iron deficiency anemia)	11,499
A09 (Other gastroenteritis and colitis of infectious and unspecified origin)	11,397
M17 (Gonarthrosis [arthrosis of knee])	10,474
L89 (Decubitus ulcer and pressure area)	10,163
I69 (Sequelae of cerebrovascular disease)	9,735
J40 (Bronchitis, not specified as acute or chronic)	9,553
R63 (Symptoms and signs concerning food and fluid intake)	9,351

© 2021 Nakanishi Y et al. *JAMA Network Open*.

G30 (Alzheimer disease)	9,351
E87 (Other disorders of fluid, electrolyte and acid-base balance)	9,348
J69 (Pneumonitis due to solids and liquids)	9,294
H26 (Other cataract)	9,193
F00 (Dementia in Alzheimer disease (G30.-†))	9,135
N39 (Other disorders of urinary system)	8,606
J30 (Vasomotor and allergic rhinitis)	8,492
J42 (Unspecified chronic bronchitis)	8,477
G62 (Other polyneuropathies)	8,438
J45 (Asthma)	8,292
F03 (Unspecified dementia)	8,031
K76 (Other diseases of liver)	7,860
N30 (Cystitis)	7,838
D64 (Other anemias)	7,738
R13 (Dysphagia)	7,490
R09 (Other symptoms and signs involving the circulatory and respiratory systems)	7,487
I49 (Other cardiac arrhythmias)	7,464
E11 (Non-insulin-dependent diabetes mellitus)	7,463
R52 (Pain, not elsewhere classified)	7,423
N40 (Hyperplasia of prostate)	7,415
I70 (Atherosclerosis)	7,244
J02 (Acute pharyngitis)	7,131
H16 (Keratitis)	6,821
Z96 (Presence of other functional implants)	6,674
I48 (Atrial fibrillation and flutter)	6,521
R60 (Oedema, not elsewhere classified)	6,410
J90 (Pleural effusion, not elsewhere classified)	6,181
R11 (Nausea and vomiting)	6,155
E79 (Disorders of purine and pyrimidine metabolism)	6,066

© 2021 Nakanishi Y et al. *JAMA Network Open*.

L98 (Other disorders of skin and subcutaneous tissue, not elsewhere classified)	5,946
M48 (Other spondylopathies)	5,836
M75 (Shoulder lesions)	5,769
R57 (Shock, not elsewhere classified)	5,751
M79 (Other soft tissue disorders, not elsewhere classified)	5,579
E88 (Other metabolic disorders)	5,567
L29 (Pruritus)	5,417
N18 (Chronic kidney disease)	5,322
F20 (Schizophrenia)	5,234
F32 (Depressive episode)	5,159
H04 (Disorders of lacrimal system)	5,058
K92 (Other diseases of digestive system)	5,021
R42 (Dizziness and giddiness)	5,000
N31 (Neuromuscular dysfunction of bladder, not elsewhere classified)	4,979
K12 (Stomatitis and related lesions)	4,958
N32 (Other disorders of bladder)	4,939
J00 (Acute nasopharyngitis [common cold])	4,851
H40 (Glaucoma)	4,689
R40 (Somnolence, stupor and coma)	4,559
N19 (Unspecified kidney failure)	4,361
S00 (Superficial injury of head)	4,325
K80 (Cholelithiasis)	4,318
E03 (Other hypothyroidism)	4,283
E56 (Other vitamin deficiencies)	4,282
K64 (Hemorrhoids and perianal venous thrombosis)	4,272
F41 (Other anxiety disorders)	4,185
H35 (Other retinal disorders)	4,183
S32 (Fracture of lumbar spine and pelvis)	4,179
S72 (Fracture of femur)	4,152

© 2021 Nakanishi Y et al. *JAMA Network Open*.

R33 (Retention of urine)	3,937
N28 (Other disorders of kidney and ureter, not elsewhere classified)	3,831
M13 (Other arthritis)	3,830
A41 (Other sepsis)	3,786
L03 (Cellulitis)	3,764
R10 (Abdominal and pelvic pain)	3,711
R50 (Fever of other and unknown origin)	3,650
H25 (Senile cataract)	3,650
M25 (Other joint disorders, not elsewhere classified)	3,643
A49 (Bacterial infection of unspecified site)	3,609
J15 (Bacterial pneumonia, not elsewhere classified)	3,479
E63 (Other nutritional deficiencies)	3,478
I46 (Cardiac arrest)	3,476
C78 (Secondary malignant neoplasm of respiratory and digestive organs)	3,459
J43 (Emphysema)	3,449
S22 (Fracture of rib(s), sternum and thoracic spine)	3,412
H01 (Other inflammation of eyelid)	3,393
U82 (Resistance to betalactam antibiotics)	3,360
I25 (Chronic ischemic heart disease)	3,298
L25 (Unspecified contact dermatitis)	3,250
F48 (Other neurotic disorders)	3,222
M19 (Other arthrosis)	3,190
C16 (Malignant neoplasm of stomach)	3,177
G40 (Epilepsy)	3,159
H61 (Other disorders of external ear)	3,132
J44 (Other chronic obstructive pulmonary disease)	3,108
D63 (Anemia in chronic diseases classified elsewhere)	3,107
R31 (Unspecified hematuria)	3,093
L08 (Other local infections of skin and subcutaneous tissue)	3,018

© 2021 Nakanishi Y et al. *JAMA Network Open*.

R51 (Headache)	3,015
G20 (Parkinson disease)	3,005
M53 (Other dorsopathies, not elsewhere classified)	2,910
H02 (Other disorders of eyelid)	2,880
R00 (Abnormalities of heart beat)	2,878
H53 (Visual disturbances)	2,833
E46 (Unspecified protein-energy malnutrition)	2,806
H36 (Retinal disorders in diseases classified elsewhere)	2,806
C34 (Malignant neoplasm of bronchus and lung)	2,763
F05 (Delirium, not induced by alcohol and other psychoactive substances)	2,727
K56 (Paralytic ileus and intestinal obstruction without hernia)	2,623
K63 (Other diseases of intestine)	2,601
R25 (Abnormal involuntary movements)	2,593
B02 (Zoster [herpes zoster])	2,550
K73 (Chronic hepatitis, not elsewhere classified)	2,544
R26 (Abnormalities of gait and mobility)	2,504
I67 (Other cerebrovascular diseases)	2,440
L50 (Urticaria)	2,415
B18 (Chronic viral hepatitis)	2,402
I74 (Arterial embolism and thrombosis)	2,393
H60 (Otitis externa)	2,384
H90 (Conductive and sensorineural hearing loss)	2,366
J32 (Chronic sinusitis)	2,359
R39 (Other symptoms and signs involving the urinary system)	2,340
J84 (Other interstitial pulmonary diseases)	2,339
L27 (Dermatitis due to substances taken internally)	2,271
F50 (Eating disorders)	2,270
I65 (Occlusion and stenosis of precerebral arteries, not resulting in cerebral infarction)	2,263
I80 (Phlebitis and thrombophlebitis)	2,193

© 2021 Nakanishi Y et al. *JAMA Network Open*.

K58 (Irritable bowel syndrome)	2,189
E53 (Deficiency of other B group vitamins)	2,187
C18 (Malignant neoplasm of colon)	2,143
D69 (Purpura and other hemorrhagic conditions)	2,126
I35 (Nonrheumatic aortic valve disorders)	2,124
S30 (Superficial injury of abdomen, lower back and pelvis)	2,114
I21 (Acute myocardial infarction)	2,108
I73 (Other peripheral vascular diseases)	2,101
I51 (Complications and ill-defined descriptions of heart disease)	2,099
K83 (Other diseases of biliary tract)	2,091
J10 (Influenza due to other identified influenza virus)	2,060
G64 (Other disorders of peripheral nervous system)	2,059
K52 (Other noninfective gastroenteritis and colitis)	2,029
Z95 (Presence of cardiac and vascular implants and grafts)	1,993
T81 (Complications of procedures, not elsewhere classified)	1,969
E83 (Disorders of mineral metabolism)	1,962
D65 (Disseminated intravascular coagulation [defibrination syndrome])	1,959
K31 (Other diseases of stomach and duodenum)	1,934
Z93 (Artificial opening status)	1,921
E77 (Disorders of glycoprotein metabolism)	1,915
S01 (Open wound of head)	1,910
I71 (Aortic aneurysm and dissection)	1,884
C79 (Secondary malignant neoplasm of other and unspecified sites)	1,884
N12 (Tubulo-interstitial nephritis, not specified as acute or chronic)	1,862
B37 (Candidiasis)	1,796
M16 (Coxarthrosis [arthrosis of hip])	1,785
H81 (Disorders of vestibular function)	1,779
N17 (Acute renal failure)	1,776
L84 (Corns and callosities)	1,725

© 2021 Nakanishi Y et al. *JAMA Network Open*.

A04 (Other bacterial intestinal infections)	1,725
C61 (Malignant neoplasm of prostate)	1,720
M06 (Other rheumatoid arthritis)	1,694
R07 (Pain in throat and chest)	1,671
F45 (Somatoform disorders)	1,668
S09 (Other and unspecified injuries of head)	1,658
M10 (Gout)	1,645
I95 (Hypotension)	1,644
S06 (Intracranial injury)	1,623
I34 (Nonrheumatic mitral valve disorders)	1,620
R19 (Other symptoms and signs involving the digestive system and abdomen)	1,599
R04 Hemorrhage from respiratory passages ()	1,589
D48 (Neoplasm of uncertain or unknown behavior of other and unspecified sites)	1,585
J11 (Influenza, virus not identified)	1,579
K86 (Other diseases of pancreas)	1,562
B00 (Herpesviral [herpes simplex] infections)	1,532
R06 (Abnormalities of breathing)	1,529
K74 (Fibrosis and cirrhosis of liver)	1,528
K85 (Idiopathic acute pancreatitis)	1,523
N20 (Calculus of kidney and ureter)	1,502
R18 (Ascites)	1,496
G46 (Vascular syndromes of brain in cerebrovascular diseases (I60-I67+))	1,484

© 2021 Nakanishi Y et al. *JAMA Network Open*.

eTable 7. Joinpoint Regression Results for Median Unadjusted Total Medical Expenditures for 1 Year Prior to Death by Age Group and Month

Age group	Segment 1		Segment 2		Segment 3		AMPC
	AR-M	MPC (95% CI), %	AR-M	MPC (95% CI), %	AR-M	MPC (95% CI), %	
Noncentenarian group							
75–79	1–4	-38.4* (-41.6 to -35.0)	4–7	-15.7* (-24.3 to -6.1)	7–12	-6.9* (-9.2 to -4.7)	-19.1* (-21.0 to -17.1)
80–84	1–4	-45.0* (-52.6 to -36.2)	4–7	-14.5 (-36.5 to 15.1)	7–12	-4.9 (-11.0 to 1.6)	-20.5* (-25.6 to -14.9)
85–89	1–4	-50.3* (-60.1 to -38.1)	4–7	-10.0 (-41.9 to 39.4)	7–12	-3.7 (-12.7 to 6.2)	-21.1* (-28.5 to -12.8)
90–94	1–3	-62.4* (-64.9 to -59.6)	3–6	-17.9* (-23.5 to -12.0)	6–12	-2.5* (-3.7 to -1.3)	-21.8* (-23.1 to -20.4)
95–99	1–3	-65.5* (-67.3 to -63.5)	3–6	-11.8* (-16.5 to -6.8)	6–12	-1.6* (-2.5 to -0.6)	-21.0* (-22.1 to -20.0)
Centenarian group							
100–104	1–3	-59.7* (-63.1 to -56.1)	3–7	-6.1* (-10.1 to -2.0)	7–12	-1.3 (-3.2 to 0.6)	-17.7* (-19.0 to -16.3)
105–109	1–3	-61.2* (-70.2 to -49.3)	3–6	-6.4 (-28.3 to 22.2)	6–12	-0.9 (-5.3 to 3.7)	-17.7* (-22.8 to -12.3)

Abbreviation: AR-M, applicable range (month); MPC, monthly percent change; AMPC, average monthly percent change.

*$P < 0.05$.

© 2021 Nakanishi Y et al. *JAMA Network Open*.

eTable 8. Joinpoint Regression Results for Median Unadjusted Hospitalization Expenditures for 1 Year Prior to Death by Age Group and Month

Age group	Segment 1		Segment 2		Segment 3		AMPC
	AR-M	MPC (95% CI), %	AR-M	MPC (95% CI), %	AR-M	MPC (95% CI), %	
Noncentenarian group							
75–79	1–6	-2.9* (-3.9 to -1.8)	6–9	-1.2 (-6.0 to 3.8)	9-12	-2.2 (-4.6 to 0.2)	-2.2* (-3.3 to -1.1)
80–84	1–5	-1.7* (-3.2 to -0.1)	5–9	-1.9 (-4.3 to 0.7)	9-12	-0.8 (-3.3 to 1.7)	-1.5* (-2.4 to -0.6)
85–89	1–5	-1.9* (-3.3 to -0.5)	5–9	-1.6 (-3.8 to 0.6)	9-12	-0.6 (-2.8 to 1.7)	-1.5* (-2.2 to -0.7)
90–94	1–3	-0.5 (-2.4 to 1.3)	3–7	-2.2* (-3.1 to -1.3)	7-12	-1.1* (-1.5 to -0.7)	-1.4* (-1.8 to -1.1)
95–99	1–3	-0.5 (-2.6 to 1.6)	3–6	-2.8* (-4.9 to -0.7)	6-12	-0.5* (-0.9 to -0.2)	-1.2* (-1.7 to -0.6)
Centenarian group							
100–104	1–3	1.8 (-3.6 to 7.4)	3-7	-2.1 (-4.7 to 0.6)	7-12	-0.9 (-2.1 to 0.3)	-0.9 (-1.9 to 0.2)
105–109	1–7	-2.8 (-5.9 to 0.4)	7-10	-5.4 (-21.8 to 14.3)	10-12	2.2 (-15.5 to 23.6)	-2.7 (-7.0 to 1.9)

Abbreviation: AR-M, applicable range (month); MPC, monthly percent change; AMPC, average monthly percent change.

*$P < 0.05$.

© 2021 Nakanishi Y et al. *JAMA Network Open*.

eTable 9. Joinpoint Regression Results for Median Unadjusted Outpatient Expenditures for 1 Year Prior to Death by Age Group and Month

Age group	Segment 1		Segment 2		Segment 3		AMPC
	AR-M	MPC (95% CI), %	AR-M	MPC (95% CI), %	AR-M	MPC (95% CI), %	
Noncentenarian group							
75–79	1–3	-5.6 (-11.7 to 0.9)	3–10	-2.3* (-3.4 to -1.2)	10–12	-3.1 (-9.3 to 3.6)	-3.1* (-4.3 to -1.8)
80–84	1–3	-9.3* (-13.3 to -5.1)	3–7	-1.4 (-3.6 to 0.9)	7–12	-1.8* (-2.8 to -0.8)	-3.1* (-3.9 to -2.2)
85–89	1–3	-14.3* (-15.3 to -13.2)	3–8	-0.5* (-0.9 to -0.1)	8–12	-1.0* (-1.4 to -0.7)	-3.4* (-3.6 to -3.1)
90–94	1–3	-20.0* (-24.3 to -15.5)	3–8	-0.8 (-2.5 to 0.9)	8–12	-0.3 (-2.0 to 1.5)	-4.4* (-5.4 to -3.5)
95–99	1–3	-23.2* (-25.0 to -21.5)	3–7	-1.3* (-2.4 to -0.1)	7–12	-0.2 (-0.7 to 0.4)	-5.2* (-5.6 to -4.8)
Centenarian group							
100–104	1–3	-29.0* (-41.7 to -13.6)	3–8	0.6 (-5.5 to 7.1)	8–12	-0.9 (-6.9 to 5.5)	-6.1* (-9.4 to -2.6)
105–109	1–3	-39.1* (-56.7 to -14.4)	3–9	1.8 (-5.7 to 9.8)	9–12	-5.9 (-20.6 to 11.6)	-9.3* (-14.7 to -3.5)

Abbreviation: AR-M, applicable range (month); MPC, monthly percent change; AMPC, average monthly percent change.

*$P < 0.05$.

© 2021 Nakanishi Y et al. *JAMA Network Open*.
